# The Chinese version of the Obsessive-Compulsive Inventory-Revised scale: Replication and extension to non-clinical and clinical individuals with OCD symptoms

**DOI:** 10.1186/1471-244X-11-129

**Published:** 2011-08-08

**Authors:** Zi-wen Peng, Wen-han Yang, Guo-dong Miao, Jin Jing, Raymond CK Chan

**Affiliations:** 1 Department of Maternal and Child Health, school of Public Health, Sun Yat-Sen University, Guangzhou, China; 2 Neuropsychology and Applied Cognitive Neuroscience Laboratory, Key Laboratory of Mental Health, Institute of Psychology, Chinese Academy of Sciences, Beijing, China; 3 Guangzhou Psychiatry Hospital, Guangzhou, China

**Keywords:** Obsessive-Compulsive Disorder, validation, Chinese

## Abstract

**Background:**

The Obsessive-Compulsive Inventory-Revised (OCI-R) was designed to evaluate the severity of obsessive-compulsive symptoms in both clinical and non-clinical samples. The aim of the study was to evaluate the psychometric properties of a Chinese version of this scale.

**Methods:**

The Chinese version of the OCI-R was administered to both a non-clinical sample (209 undergraduate students) and a clinical sample (56 obsessive-compulsive disorder (OCD) patients). Confirmatory factor analysis was conducted to examine the construct validity of the OCI-R in the non-clinical sample. The internal consistency at baseline and test-retest reliabilities at 4-week interval was examined in both the non-clinical and clinical samples.

**Results:**

The confirmatory factor analysis of the non-clinical sample confirmed a 6-factor model suggested by the original authors of the instrument (df = 120, RMSEA = 0.068, CFI = 0.88, NNFI = 0.85, GFI = 0.89). The internal consistency and test-retest reliability were at an acceptable range for both the non-clinical and clinical samples. The OCI-R also showed good clinical discrimination for patients with OCD from healthy controls.

**Conclusions:**

The Chinese version of the OCI-R is a valid and reliable instrument for measuring OCD symptoms in the Chinese context.

## Background

Obsessive-compulsive disorder (OCD) is a chronic psychiatric illness with a mean lifetime prevalence of 2% to 3% in the general population [1]. OCD is characterized by intense anxiety caused by unwanted, intrusive, persistent thoughts, images, or impulses (obsessions), leading to repetitive behaviours or mental acts (compulsions) that the patient feels driven to perform to prevent or reduce his or her distress or anxiety [2]. Several self-reporting questionnaires evaluating the severity of OCD have been developed, such as the Yale Brown Obsessive-Compulsive Scale [3], the Paudua Inventory [4], the Maudsley Obsessive-Compulsive Inventory [5], the Vancouver Obsessional Compulsive Inventory [6], and the Schedule of Compulsions, Obsessions, and Pathological Impulses [7]. However, these interview-based measures can be time-consuming and expensive, requiring interviewer training and establishment of interviewer reliability. These constraints may preclude their use in many clinical settings.

For these reasons, a comprehensive yet brief self-report measure of the symptoms of OCD would be advantageous. The Obsessive-Compulsive Inventory was specifically developed to measure the intensity of the various symptoms that characterize OCD, assess their frequency and the distress they caused during the previous month, as well as estimating the overall severity of the disorder [8]. The original OCI consists of seven subscales, namely Washing (eight items), Checking (nine items), Mental Neutralizing (six items), Obsessing (eight items), Ordering (five items), Hoarding (three items), and Doubting (three items). It is rated on a five-point Likert scale to assess the frequency of symptoms and the associated distress. The original version of the OCI has been demonstrated to have good to excellent internal consistency for the full scale and the subscales (r ranged from 0.59 to 0.96), good test-retest reliability for non-clinical samples (r ranged from 0.68 to 0.90) and clinical samples (r ranged from 0.77 to 0.97), and excellent discriminant validity and satisfactory convergent validity [8]. However, the utility of this scale in clinical setting was limited by its length.

Recently, a shorter, 18-item version of the Obsessions and Compulsions Inventory Revised (OCI-R) was developed by Foa et al [9]. This revised version was found to have a stable factor structure, high internal consistency for the full scale (ranged from 0.81 to 0.93) and for the subscales (ranged from 0.65 to 0.90), good to excellent test-retest reliability (coefficient r ranged from 0.57 to 0.91), good discriminant validity and satisfactory convergent validity. It has been shown to discriminate patients with OCD from non-clinical samples as well as from patients with anxiety disorder [9]. In summary, the OCI and the OCI-R were shown to have good psychometric properties in both clinical and non-clinical samples, and have been validated with different versions [10-16]. However, all of these validations were done in western samples only.

The purpose of this study was to validate a Chinese version of the OCI-R in a non-clinical sample and a clinical sample with OCD. The availability of the OCI-R in Chinese would definitely provide researchers with additional information to assess the severity of OCD symptoms in the Chinese context, and to facilitate cross-cultural comparison in the near future.

## Methods

### Participants

The non-clinical sample consisted of 209 undergraduate student volunteers (116 females, 93 males), recruited from the Sun-Yat Sen University in Guangzhou, China. The mean age and number of years of education was 20.17 years (SD = 2.06) and 15.32 years (SD = 1.25), respectively. Fifty-six patients with OCD (15 females, 41 males) were recruited from the out-patient clinic of the Guangzhou Psychiatric Hospital. The mean age and number of years of education was 24.36 years (SD = 3.08) and 12.59 years (SD = 3.21), respectively. All of them met the diagnostic criteria of the Diagnostic and Statistical Manual of Mental Disorders (DSM-IV) [17], and were confirmed by a clinical interview utilizing the Yale-Brown Obsessive-Compulsive Scale (Y-BOCS) [3]. A subgroup of 41 non-clinical participants (18 females, 33 males) and 27 patients with OCD (five females, 22 males) were invited to complete a test-retest session at four weeks later.

Potential non-clinical and clinical participants were excluded if they were: (1) aged under18 or over 50 years; (2) had a history of head injury, central nervous system diseases, or mental illness (except OCD patients); (3) had a history of substance abuse. These exclusion criteria ensured that all the participants could understand the procedures in the study. Moreover, all OCD participants were excluded if they had comorbid generalized anxiety disorder or major depression.

### The Chinese version of the OCI-R

The OCI-R is a self-reporting questionnaire consisting of 18 items evaluating OCD symptoms. The revised version has six subscales, each containing three items: Washing (5, 11, 17), Obsessing (6, 12, 18), Hoarding (1, 7, 13), Ordering (3, 9, 15), Checking (2, 8, 14) and Neutralising (4, 10, 16). Respondents are requested to self-report to what degree the situation describe in each particular statement distresses them during the past month on a five-point scale (0 = not at all; 4 = extremely). Total score may range from 0 to 72 [9].

The validation of the Chinese version of OCI-R followed the international guidelines suggested by Beaton for cross-cultural validation of self-reported measures, namely (1) the initial translation of the original scale into the used language, (2) synthesis of conceptions, (3) back-translation, (4) expert committee review on the relevance and representation of items used for the final outcome setting, and (5) pilot testing and probe to get at understanding of item [18]. After having received authorization from the author of the instrument, it was translated into Chinese. The translation from English into Chinese was done by two bilingual psychiatrists who had never seen the original scales to ensure their impartiality. Then, the translations of both psychiatrists were compared and merged, resulting in an initial Chinese version of the OCI-R. The initial version was administered to 25 OCD in-patients at the Guangzhou Psychiatric Hospital. The main purpose was to verify if patients could understand the various items of the questionnaires. At this time, all suggestions provided by the patients were taken into account, and adjustments were made wherever necessary. Once all items in the Chinese version of the OCI-R were considered adequate for use, they were back-translated into English by another bilingual psychiatrist who was not involved in the previous translation process.

### Measures of psychopathology

The Y-BOCS was administered to each participant to assess the severity of OCD symptoms and to provide a measure of concurrent validity of the translated OCI-R. The Y-BOCS is a commonly used clinical interview for OCD patients. Severity scores (obsessions, compulsions, and their sum) are derived from 10 items, each rated on a five-point scale [3]. The SCID-IV [19] was also conducted by experienced psychiatrists in the Guangzhou Psychiatric Hospital to determine DSM-IV diagnoses.

### Procedure

All participants were administered the Chinese version of the OCI-R. The procedures of this study were approved by the ethics committee of the Guangzhou Psychiatric Hospital, where the project was based. Written consent was obtained from all the participants before the assessment.

### Data analysis

A confirmatory factor analysis was performed to examine the latent structure of the Chinese version of the OCI-R in the non-clinical sample. We computed a six-factor confirmatory analysis using the program Proc Calis in SAS Version 8.02. As in the original instrument, we used the criteria recommended by Hu and Bentler [20]. The goodness of fit was evaluated by the following criteria: chi-square χ^2 ^Satorra-Bentler, the ratio between chi-square and degrees of freedom (χ^2^/d.f.)≦3, root mean square error of approximation (RMSEA)≦0.08; non-normed fit index (NNFI)≥0.95, and comparative fit index (CFI) ≥0.95.

Cronbach's alphas and correlation coefficients were computed to determine the internal consistency and test-retest reliability of the Chinese version of the OCI-R, respectively. Moreover, concurrent validity of the OCI-R was also determined using Pearson correlations with Y-BOCS scores and comparison of the OCI-R scores between patients with OCD and matched healthy controls.

## Results

### Confirmatory Factor Analysis

**Figure 1:**
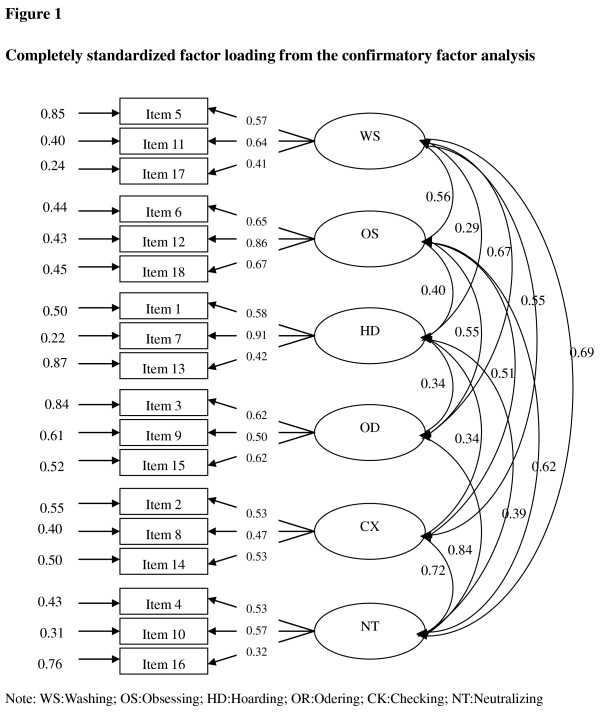
**Completely standardized factor loading from the confirmatory factor analysis**.

The six-factor solution showed a chi-square of 231.36 (df = 120, N = 209), a Root Mean Square Error of Approximation (RMSEA) of 0.068, a Comparative Fit Index (CFI) of 0.88, a Non-Normed Fit Index (NNFI) of 0.85, and a Goodness of Fit Index (GFI) of 0.89. According to Schermelleh-Engel and Moosbrugger [21], all these indices suggested a good fit for the model and thus confirmed the six-factor structure. The standardized factor loadings of the confirmatory factor analysis are shown in Figure 1.

### Internal Consistency

The Cronbach alpha for the OCI-R total scale was 0.84 indicating an excellent internal consistency. Even though the majority of the coefficient alphas for the subscales were lower than those reported by Foa et al [9], they nevertheless had an acceptable range of internal consistency (Washing = 0.64, Obsessing = 0.77, Hoarding = 0.66, Ordering = 0.63, Checking = 0.61, Neutralizing = 0.53). Inter-item correlations between subscales and the total score for the non-clinical group revealed significant and large effects (coefficient r ranged from 0.58 to 0.74; p < 0.05). However, the inter-item correlations between subscales were modest (coefficient r ranged from 0.19 to 0.49; p < 0.05). For the 56 OCD patients, the internal consistency and inter-item correlation of the total score on the OCI-R was 0.89 and 0.82, respectively, whereas the internal consistency and inter-item correlation for the subscales ranged from 0.64 to 0.83 and 0.73 to 0.94, respectively.

### Test-retest Reliability

For the non-clinical sample, the test-retest reliability for the total score of the OCI-R was excellent (r = 0.96) and good to excellent for subscales (Washing = 0.58, Obsessing = 0.39, Hoarding = 0.48, Ordering = 0.62, Checking = 0.44, Neutralizing = 0.57). For the clinical sample, the test-retest reliability was also good to excellent for the total score (0.82) and subscales (Washing = 0.78, Obsessing = 0.94, Hoarding = 0.73, Ordering = 0.91, Checking = 0.74, Neutralizing = 0.82).

### Concurrent Validity

The correlation coefficient between the OCI-R and the Y-BOCS for OCD patients was 0.34. Table 1 also shows that patients with OCD demonstrated significantly higher prevalence of OCD-R total score and subscale scores (except Hoarding) than matched healthy controls.

**Table 1:** Means and Standard Deviations for subscales and total scores

	HC(n = 209)	OCD(n = 56)	t-value
Washing	2.23(2.03)	3.88(3.12)	4.74**
Checking	2.42(1.93)	4.07(2.99)	5.00**
Ordering	3.53(2.29)	3.68(2.89)	0.40**
Obsessing	3.17(2.45)	5.39(3.20)	5.62**
Hoarding	3.14(2.23)	2.39(2.56)	-2.15
Neutralizing	2.04(1.84)	3.11(2.66)	3.47**
Total score	16.53(8.76)	22.52(12.43)	4.10**

Note: HC: healthy control (non-clinical sample); OCD: Obsessive-Compulsive Disorder patients. *P < 0.05, **P < 0.01 (two-detailed).

## Discussion

The aim of this study was to validate a Chinese version of the OCI-R in both non-clinical and clinical samples. The confirmatory factor analysis confirmed the six-factor structure of the OCI-R, namely washing, checking, ordering, obsessing, mental neutralizing, and hoarding. The results suggest that the Chinese version of the OCI-R has a good fit for the model of the original and other western versions of OCI-R developed by Foa et al [9]. More importantly, our study further confirmed that the structure of OCD symptoms is similar across different cultures [10-16].

The Chinese version of the OCI-R demonstrated good internal consistencies and test-retest reliabilities for both healthy controls and OCD patients. Moreover, the translated OCI-R was significantly associated with the Y-BOCS showing an acceptable concurrent validity of measuring OCD symptoms in the Chinese setting. Our study also showed that the Chinese version of the OCI-R had good clinical discriminatory properties between patients with OCD and healthy controls. The non-significant finding in hoarding symptoms is also consistent with previous studies using the OCI-R in Western samples. For example, Huppert et al. [10] found that the scores of patients with OCD were no more elevated on this subscale than non-clinical participants. Our finding that the neutralizing subscale had the lowest internal consistency is also consistent with previous studies [8-10]. Huppert et al. [10] suggested that the three items captured by the neutralizing subscale seem to tap into relatively different concepts (bad numbers vs. counting vs. repeating numbers). Future research should consider an expansion of items for neutralizing behaviour.

However, it should be noted that there are some discrepancies between our results and the original as well as the other translated versions of the OCI-R. Although the latent factor structure, reliability, and validity of the subscales of the OCI-R in our study are consistent with previous validation work done in other countries [10-16], our samples had lower mean scores and Cronbach's alpha coefficients than other validation studies of OCI-R in non-clinical samples [12-14]. This might be due to several reasons including the source of the non-clinical sample, the relatively small sample size, and potential cultural impact of reporting OCD symptoms in our current sample. Moreover, our clinical sample also reported a higher Y-BOCS score than other clinical samples [10,22]. This might again be due to the cultural differences. However, given the small and non-representative sample of our current findings, this speculation should be confirmed by a larger sample size in the near future

## Conclusions

Taken together, our findings have demonstrated a stable factor structure of OCD symptoms in Chinese clinical and non-clinical samples, and that the Chinese version of the OCI-R exhibits satisfactory psychometric properties. Our findings support that this version of the OCI-R is ecologically and culturally valid in the Chinese context.

## Abbreviations

CFI: comparative fit index; CK: Checking; DSM- IV: Diagnostic and Statistical Manual of Mental Disorders criteria fourth edition; GFI: Goodness of Fit Index; HD: Hoarding; MOCI: the Maudsley Obsessive-Compulsive Inventory; NNFI: non-normed fit index; NT: Neutralizing; OCD: Obsessive-Compulsive disorder; OCI: Obsessive-Compulsive Inventory; OCI-R: Obsessive-Compulsive Inventory-Revised; OR: Ordering; OS: Obsessing; PI: the Paudua Inventory; RMSEA: Root Mean Square Error Approximate; ROC: Receiver Operating Characteristic analyses; SCID-CV: Structured clinical interview for DSM-IV axis I disorders, clinician version; SCOPI: the Schedule of Compulsions, Obsessions, and Pathological Impulses; VOCI: the Vancouver Obsessional Compulsive Inventory; WS: Washing; Y-BOCS: Yale-Brown Obsessive-Compulsive scale.

## Competing interests

The authors declare that they have no competing interests.

## Authors' contributions

ZWP, GDM and JJ designed the study, collected and analyzed the data, and wrote the first draft of the paper. WHY collected the data and assisted data analysis. RCKC conceived and designed the study, and wrote the first draft of the paper. All authors read and approved the final manuscript.

## Pre-publication history

The pre-publication history for this paper can be accessed here:

http://www.biomedcentral.com/1471-244X/11/129/prepub
